# A Novel Retro-Inverso Peptide Inhibitor Reduces Amyloid Deposition, Oxidation and Inflammation and Stimulates Neurogenesis in the APPswe/PS1ΔE9 Mouse Model of Alzheimer’s Disease

**DOI:** 10.1371/journal.pone.0054769

**Published:** 2013-01-31

**Authors:** Vadivel Parthsarathy, Paula L. McClean, Christian Hölscher, Mark Taylor, Claire Tinker, Glynn Jones, Oleg Kolosov, Elisa Salvati, Maria Gregori, Massimo Masserini, David Allsop

**Affiliations:** 1 School of Biomedical Sciences, University of Ulster, Coleraine, Co. Londonderry, United Kingdom; 2 Division of Biomedical and Life Sciences, University of Lancaster, Lancaster, Lancashire, United Kingdom; 3 Department of Physics, University of Lancaster, Lancaster, Lancashire, United Kingdom; 4 Department of Experimental Medicine, University of Milano-Bicocca, Monza, Milan, Italy; Cambridge Institute for Medical Research, United Kingdom

## Abstract

Previously, we have developed a retro-inverso peptide inhibitor (RI-OR2, rGffvlkGr) that blocks the *in vitro* formation and toxicity of the Aβ oligomers which are thought to be a cause of neurodegeneration and memory loss in Alzheimer’s disease. We have now attached a retro-inverted version of the HIV protein transduction domain ‘TAT’ to RI-OR2 to target this new inhibitor (RI-OR2-TAT, Ac-rGffvlkGrrrrqrrkkrGy-NH_2_) into the brain. Following its peripheral injection, a fluorescein-labelled version of RI-OR2-TAT was found to cross the blood brain barrier and bind to the amyloid plaques and activated microglial cells present in the cerebral cortex of 17-months-old APPswe/PS1ΔE9 transgenic mice. Daily intraperitoneal injection of RI-OR2-TAT (at 100 nmol/kg) for 21 days into 10-months-old APPswe/PS1ΔE9 mice resulted in a 25% reduction (p<0.01) in the cerebral cortex of Aβ oligomer levels, a 32% reduction (p<0.0001) of β-amyloid plaque count, a 44% reduction (p<0.0001) in the numbers of activated microglial cells, and a 25% reduction (p<0.0001) in oxidative damage, while the number of young neurons in the dentate gyrus was increased by 210% (p<0.0001), all compared to control APPswe/PS1ΔE9 mice injected with vehicle (saline) alone. Our data suggest that oxidative damage, inflammation, and inhibition of neurogenesis are all a downstream consequence of Aβ aggregation, and identify a novel brain-penetrant retro-inverso peptide inhibitor of Aβ oligomer formation for further testing in humans as a potential disease-modifying treatment for Alzheimer’s disease.

## Introduction

Alzheimer’s disease (AD) is the leading cause of dementia in the elderly and afflicts around 12% of people over the age of 65, rising to 46% of those over the age of 80 [1]. In 2010 there were 36 million cases of AD worldwide, and this number is expected to approximately double every 20 years, to 66 million in 2030, and 115 million in 2050 [2]. Despite substantial progress in understanding the pathobiology of AD, this knowledge has not been translated yet into any effective treatment. Currently available drugs for AD can only temporarily alleviate the symptoms of the disease, and they do not have a major impact on disease progression. It is, therefore, imperative that new and more effective treatments are developed.

There is substantial and well known evidence from molecular genetics, transgenic animal studies and aggregation/toxicity studies to suggest that the conversion of the β-amyloid (Aβ) peptide from monomers into aggregated forms in the brain is a key (and possibly seminal) event in the pathogenesis of AD [3], [4]. The other major characteristic pathological change in AD is the formation of neurofibrillary tangles (NFTs) inside nerve cells, which are derived from a hyperphosphorylated form of tau protein. NFTs are likely to represent a secondary feature, following on from the deposition of Aβ [5]. Aggregated forms of Aβ are toxic to nerve cells, and have potent effects on memory and learning. There is increasing emphasis on ‘soluble oligomers’ as the pathological form of Aβ, rather than amyloid fibres, and these small oligomers could be one of the major causes of neurodegeneration and memory loss in AD [6]–[12]. Inhibition of toxic Aβ oligomer formation is therefore a potential therapeutic target for AD and inhibitors of early-stage Aβ aggregation could slow or even halt the progression of this disease [6]-[15]. However, the downstream consequences of inhibition of Aβ oligomer formation *in vivo* have not been clearly established. Although considerable progress has been made in discovering a wide range of inhibitors of Aβ aggregation, many of these past studies have utilized techniques such as turbidity, thioflavin-T binding, sedimentation and Congo red binding, which can only identify compounds that are capable of inhibiting the formation of fibrils, and so the effects of these inhibitors on oligomer formation are often not clear. Furthermore, most of these inhibitors are not suitable for clinical development, and so very few of them have succeeded in animal studies or have progressed to human clinical trials [15].

Some of us have reported previously the development of a retro-inverso peptide inhibitor (RI-OR2, Ac-rGffvlkGr-NH_2_) that blocks the formation of Aβ oligomers and fibrils *in vitro* and also inhibits the toxic effects of Aβ on cultured cells [16]. This inhibitor consists of a retro-inverted version of the internal Aβ(16–20) sequence, KLVFF, flanked by the solubilizing residues rG- and -Gr. In the retro-inverso peptide, the L-amino acids are replaced by D-amino acids (represented by lower case letters) and the sequence is reversed. KLVFF was chosen because it is the region that is primarily responsible for the self-association and aggregation of Aβ [17], [18]. Other groups have also developed peptide-based inhibitors of Aβ aggregation [19]-[26]. For example, Soto and co-workers have designed ‘β-sheet breaker peptides’ by incorporating proline residues into a similar part of the Aβ peptide sequence [20], [21] and another strategy has used N-methylated peptides [22], [23]. Although there has been considerable research into these types of peptide inhibitors, none of them have entered into advanced human clinical trials. Our own inhibitor, RI-OR2, was shown to be highly resistant to proteolysis, and so should be stable *in vivo* [16]. However, in surface plasmon resonance (SPR) experiments, it was found to bind to immobilized Aβ(1–42) (Aβ42) monomers, oligomers and fibrils with only modest affinity (k_d = _9–29 µM) [16]. Moreover, RI-OR2 is not designed to penetrate the blood-brain barrier (BBB) and so is unlikely to be a suitable drug candidate for AD.

For this report, we have attached a retro-inverted version of the HIV protein transduction domain ‘TAT’ [27] to RI-OR2, to produce a cell-permeable and brain-penetrant inhibitor of Aβ oligomer and fibril formation (Ac-rGffvlkGrrrrqrrkkrGy-NH_2_) which we refer to as RI-OR2-TAT. We show that peripheral administration of this inhibitor into the APPswe/PS1ΔE9 (APP/PS1) transgenic mouse model of AD reduced Aβ oligomer formation, amyloid deposition and the associated oxidation and inflammatory reactions in the brain, and also had a marked effect on stimulation of neurogenesis.

## Methods

### Ethics Statement

All animal experiments were licensed by the UK Home Office in accordance with the UK animals (Scientific Procedures) Act 1986.

### Peptides

All of the inhibitors used for this study were custom made by Cambridge Peptides (Birmingham, UK) and were >95% purity. Recombinant Aβ42, Ultrapure, was purchased from rPeptide, Bogart, Georgia, USA. Prior to use for *in vitro* aggregation experiments, Aβ42 was deseeded [16]. The peptide was dissolved at 1 mg/ml in trifuoroacetic acid containing 4.5% thioanisol. After 1 h incubation at room temperature, all liquid was evaporated using a stream of oxygen-free nitrogen gas. The peptide was dissolved in 1,1,1,3,3,3-hexafluoro-2-propanol (HFIP) and this was then removed by centrifugation under vacuum. The latter process was repeated twice more before splitting the solution into smaller aliquots which were dried and stored at −20°C until use.

### Thioflavin T (ThT) Assay for Aβ Fibrils

This method was the same as that published previously [16]. Briefly, 60 µl samples of solutions containing Aβ42 (25 µM), ThT (15 µM) and between 0 and 125 µM RI-OR2 or RI-OR2–TAT, in 10 mM phosphate buffered saline (PBS), pH 7.4, were incubated in sealed, black, clear-bottomed 384-well plates (Corning). ThT fluorescence was monitored every 10 mins (λex = 442 nm, λem = 483 nm) for 48 h at 30°C in a Biotek Synergy 2 plate reader.

### Immunoassay for Aβ Oligomers (*in vitro*)

This is a more sensitive test than the ThT assay for the detection of early oligomeric forms of Aβ [16]. Microtitre plates (96-well, Maxisorb) were coated with 6E10, diluted 1∶1000 in 10 mM PBS, pH 7.4, for overnight at 4°C. The plates were blocked with assay buffer (Tris-buffered saline (TBS) (pH 7.4), containing 0.05% γ-globulins and 0.005% Tween 20) plus 5% gelatine for 1 h at 37°C. Samples of peptide (12.5 µM Aβ42 and 0, 3.125, 6.25, 12.5, 25 or 62.5 µM RI-OR2–TAT in 10 mM PBS, pH 7.4) were incubated at 25°C for 0, 4, 8, 24 or 48 h, diluted to 1 µM Aβ, and plated in triplicate. The plates were incubated for 1 h at 37°C and then washed with PBS plus 0.05% Tween-20 (PBST). A 1∶1,000 dilution of biotinylated 6E10 was then added to each well, left for 1 h at 37°C, and the plates were washed with PBST. Europium-linked streptavidin was then added to the wells at 1∶500 dilution in StrepE buffer (TBS containing 20 µM DTPA, 0.5% bovine serum albumin, and 0.05% γ-globulins), incubated for 1 h, and washed as before. Enhancer solution was added and the plates were read on a Wallac Victor 2 plate reader, using the time-resolved fluorescence setting for europium.

### Cell Penetration and Cytotoxicity Experiments

For cell penetration experiments, the fluorescein-tagged peptides were added to cell growth medium on a slide containing cultured SHSY-5Y cells at a concentration of 0.1 µM and micrographs were taken after 10 mins (Flu-RI-OR2-TAT) or 1 h (Flu-RI-OR2) incubations.

For cell toxicity assays, SHSY-5Y neuroblastoma cells were maintained in 10% Fetal Calf Serum supplemented Dulbecco’s Modified Eagle Medium (DMEM, Gibco) under standard mammalian cell culture conditions. The cells were transferred to sterile 96-well growth plates at 20,000 cells/well and 4 wells per condition. To test for any toxic effects of RI-OR2–TAT alone on the cells, they were left to adhere for 24 h before the addition of the inhibitor (at 12.5, 25, 50, 100 and 200 µM), in DMEM. To assess the ability of RI-OR2–TAT to protect against Aβ toxicity, the cell growth medium was changed to Optimem (Invitrogen) and Aβ42, pre-aggregated (at 100 µM) for 24 h at 25°C in PBS, was added to the cells at a concentration of 5 µM, together with various amounts (0, 0.1, 0.5, 1, 5 or 10 µM) of RI-OR2–TAT. In each case, after a further 24 h incubation period, the viability of the cells was assessed using a CytoTox 96® Non-Radioactive Cytotoxicity Assay (LDH) Assay (Promega) kit.

### Surface Plasmon Resonance (SPR)

These experiments were conducted using a Sensi Q semi-automatic SPR machine (ICx Nomadics). This apparatus has two parallel flow cells; one was used to immobilize Aβ42 fibrils while the other was used as “reference” (empty surface). A COOH5 sensor chip (ICx Nomadics) was employed for this purpose and the peptide was immobilized by amine coupling chemistry. Briefly, after surface activation, the peptide preparation was diluted to 10 µM in acetate buffer (pH 4.0) and then injected for 5 min at a flow rate of 30 µl/min. Any remaining activated groups were blocked with ethanolamine (pH 8.0). The final immobilization level was ∼5,000 resonance units (1 RU = 1 pg of protein/mm^2^). The empty “reference” surface was prepared in parallel using the same immobilization procedure, but without addition of the peptide. Sensorgrams were then obtained *via* injection of three different concentrations of RI-OR2–TAT (1, 3 and 6 µM), as well as the vehicle (PBS with 0.005% Tween 20), over the immobilized ligand or control surface, in parallel.

These SPR data can be interpreted to provide an estimate for the affinity of binding of the inhibitors to Aβ fibrils [16], [28]. Data for the three concentrations of RI-OR2-TAT were fitted separately by Qdat software [ICx Nomadics] using the 1∶1 pseudo-first order Langmuir interaction model. Eq. 1 was used to fit the association while the dissociation was fitted by eq. 2. The rate constants (k_on_ and k_off_) were used to calculate k_d_ as reported in eq. 3.
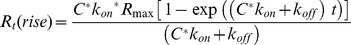
(1)

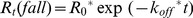
(2)

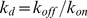
(3)


Where R_t_ is binding response at time t; C is the molar concentration of the analyte at the interaction surface at time t; R_max_ is the maximal binding response; R_0_ is the binding response at time t_0_ (i. e. time at the onset of the dissociation phase); k_on_ is the association rate constant; k_off_ is the dissociation rate constant. Values were expressed as a mean ± SD.

### Atomic Force Microscopy (AFM)

Aβ42 was incubated at 25 µM in the presence or absence of 12.5 µM RI-OR2-TAT in PBS, pH 7.4, for 24 h. Samples were diluted 1∶10 in PBS and then a 2 µl aliquot was deposited onto the surface of freshly cleaned mica and allowed to dry.

Images were obtained in tapping mode using a Multimode™ SPM NanoScope IIIa microscope (Digital Instruments, New York, USA). Silicon cantilever tips measuring 125 µm long, 30 µm wide and with a tip radius <10 nm were used (Budget Sensors, Bulgaria). The resonance frequency was 300 kHz and force constants 40 N/m. All images were first order flattened and edited using WSxM 5.0 Develop 4.3 software, (Nanotech, Madrid, Spain) [29].

### Animals

APPswe/PS1ΔE9 mice with a C57Bl/6 background (APP/PS1 mice) were obtained from the Jackson lab (http://research.jax.org/repository/alzheimers.html). To determine if Flu-RI-OR2-TAT crosses the blood brain barrier (experiment 1), 2 male 17-months-old APP/PS1 animals and 2 16-months-old C57BL/6 animals were used. To look at the effects of RI-OR2-TAT on mouse brain pathology (experiment 2), 8 female 10-months-old APP/PS1 mice were used. The animals were housed in single cages in a temperature controlled holding room (21.5°C±1) with 12∶12 h light and dark cycle. Food and water was available *ad libitum*.

### Drug Treatment

For experiment 1, all 4 animals were injected i.p. with 100 nmol/kg of Flu-RI-OR2-TAT in 0.9% NaCl at 0 h. The animals were then housed in a dark room. After 1 h, the animals were anaesthetised with isoflurane and sodium barbiturate (Dolethal, Bayer, Germany), transcardially perfused and the brains were retrieved and fixed in 4% ice cold paraformaldehyde.

For experiment 2, 4 animals were injected intraperitonially (i.p.) with 0.9% NaCl (vehicle control) and the other 4 with RI-OR2-TAT (100 nmol/kg in 0.9% NaCl) once daily, for 21 days. On the 22^nd^ day, the animals were perfused transcardially with ice-cold PBS and 4% paraformaldehyde and then the brains were removed and post-fixed in 4% ice cold paraformaldehyde.

### Immunostaining

All brains in paraformaldehyde were transferred to 30% sucrose overnight and then snap frozen with Envirofreez™ (Sigma, UK) and, using a Leica cryostat, 40 microns thick coronal sections were cut at anatomical regions of −2 to −3 bregma. Sections were preserved in cryoprotect with the first section taken at random and then every 5^th^ section afterwards.

For experiment 1 (BBB penetration), individual sections were incubated with the following primary antibodies at 4°C overnight: anti-Aβ (1∶200 dilution, Invitrogen, 71–5800), or anti-Iba 1 (1∶1000 dilution, Wako 016-20001, Germany). Sections were then stained with goat anti-rabbit Alexa Fluor 555 (1∶150 dilution, A21428 Invitrogen) for 1 h.

For experiment 2 (effects on brain pathology), sections were examined for activated microglia (using anti-Iba-1 antibody), oxidative stress (anti-8-oxo-guanine antibody), immature neurons (anti-doublecourtin antibody) or total plaque load (anti-Aβ antibody). Activated microglia were visualised following incubation of brain sections in 0.05 M sodium citrate buffer, pH 9 at 90°C for 30 minutes before they were stained using an anti Iba-1 antibody (1∶2000 dilution, Wako 016-20001, Germany) [30]. Oxidative stress was assessed by denaturing DNA in each section using 2N HCl and the sections were then stained for 8-oxo guanine (1∶200 dilution, MAB3560, Millipore). Young immature neurons were detected by immunostaining for doublecortin (1∶200 dilution, sc-66911, Santa Cruz Biotechnology). Total plaque load was quantified by immunostaining for β-amyloid (1∶200 dilution, Invitrogen, 71–5800).

All sections were visualized using an Axio Scope 1 fluorescence microscope (Zeiss, Germany). For quantification, two images per section were taken from the cortex area with a 10X objective for 8-oxo guanine and β-amyloid staining, and a 100X objective for Iba1 staining, with a minimum of 5 sections visualized per animal. Image analysis was performed using multi threshold plug in Image J (NIH, USA).

For experiment 1, sections were visualized using multichannel filters and images were merged using the microscope software.

### ELISA Assay for Aß Oligomer Analysis

Human oligomerised Aβ was measured using a kit purchased from Invitrogen, according to the manufacturer’s instructions. Briefly, the right hemispheres from the brains of transgenic APP/PS1 mice treated with RI-OR2-TAT, or controls, were pooled separately and homogenized. Quantification of total protein was determined using the Bradford protein assay and was measured on a Nanophotometer (Implen, Germany). The homogenates were centrifuged at 100,000 g at 4°C for 1 h. The supernatant was then diluted 1∶10 before carrying out the sandwich ELISA, which measures soluble Aβ oligomer levels, but not amyloid monomers, as detailed in the manufacturer’s protocol. The final Aβ oligomer values were determined following normalization of total protein levels.

### Statistics

The data are presented as either mean ± standard deviation (*in vitro* aggregation and cell culture experiments) or mean ± standard error of the mean (*in vivo* experiments). Treated versus control conditions were compared using a student t-test and a *p*-value of <0.05 was considered to be statistically significant.

## Results

### RI-OR2-TAT Inhibits the Formation of Aβ42 Oligomers and Fibrils *in vitro*

**Figure 1:**
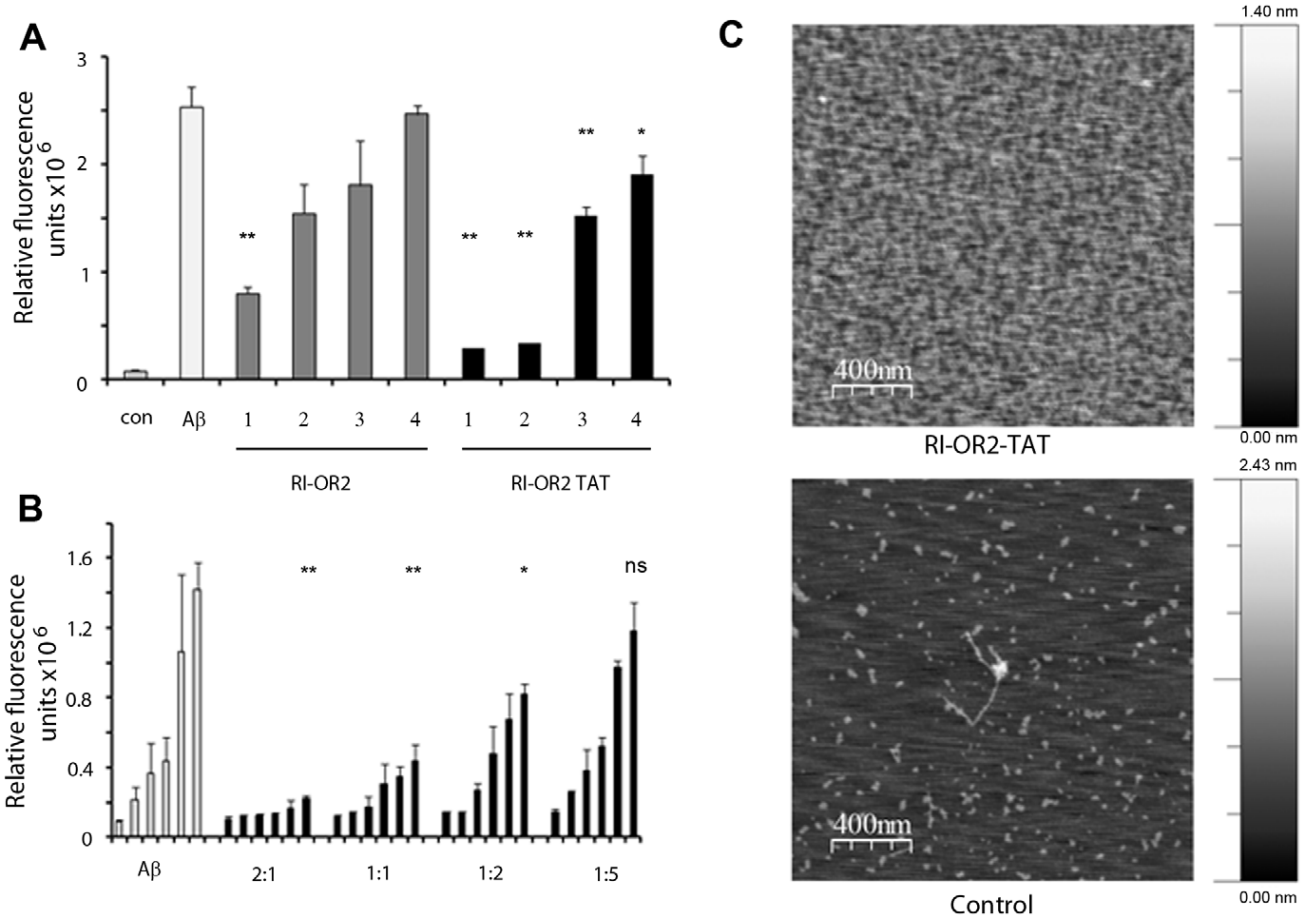
RI-OR2 and RI-OR2-TAT inhibit the aggregation of Aβ42. (**A**) ThT data for Aβ42 incubated with and without these inhibitors: ‘con’ is buffer only; ‘Aβ’ is Aβ42 incubated at 25 µM for 48 h, with no inhibitor; the dark grey bars are Aβ42 incubated for 48 h with RI-OR2 at 1–25∶25, 2–12.5∶25, 3–6.75∶25, 4–2.5∶25 µM concentration of inhibitor:Aβ; and the black bars are Aβ42 incubated with RI-OR2-TAT at these same molar ratios. (**B**) Effects of the inhibitors on detection of multimeric Aβ42 by immunoassay. The data for Aβ42 alone, incubated at 12.5 µM, are shown on the left (pale bars), and for Aβ42 incubated with 25∶12.5, 12.5∶12.5, 6.75∶12.5 and 2.5∶12.5 µmolar concentrations of RI-OR2-TAT:Aβ on the right (black bars). In each case, the consecutive bars are for 0, 4, 8, 24 and 48 h incubations. Statistics: for both (**A**) and (**B**), *denotes p<0.05 for treated sample versus untreated control, and **denotes p<0.01. (**C)** AFM images of a 24 h incubation of Aβ42 (25 µM) in the presence and absence (Control) of RI-OR2-TAT (12.5 µM). Scale bar is to the right.

In a thioflavin T (ThT) assay, which detects mainly amyloid fibrils, the presence of RI-OR2-TAT resulted in lower fluorescence after 48 h incubation of Aβ42 at molar ratios of 1∶1, 1∶2, 1∶4 and 1∶10 (inhibitor:Aβ42) and seemed to be a slightly better inhibitor than RI-OR2 (**Fig. 1A**). These inhibitors were also tested in an immunoassay technique for the detection of early-stage Aβ oligomers [16]. This assay uses monoclonal anti-Aβ antibody 6E10 to capture Aβ from solution and a biotinylated form of 6E10 as the detection antibody, in a sandwich system. Monomeric Aβ has only a single 6E10 epitope, which is occupied by the capture antibody, and so it cannot subsequently bind to the detection antibody. On the other hand, multimeric Aβ has binding sites available for both capture and detection, giving rise to a strong immunoassay signal. In Aβ aggregation time-course experiments, this assay produces a signal before the ThT method, when only small oligomers are present [16]. RI-OR2-TAT considerably reduced the development of an immunoassay signal, at 2∶1 and 1∶1 molar ratios of inhibitor:Aβ42, even at the earliest time points where such a signal was detectable (4 and 8 h), indicating inhibition of oligomer formation (**Fig. 1B**). This was confirmed by atomic force microscope (AFM) images (**Fig. 1C**), which showed clear inhibition of oligomer formation at a 1∶2 molar ratio of RI-OR2-TAT:Aβ42.

### RI-OR2-TAT has an Increased Binding Affinity for Aβ42 Compared to RI-OR2

**Figure 2:**
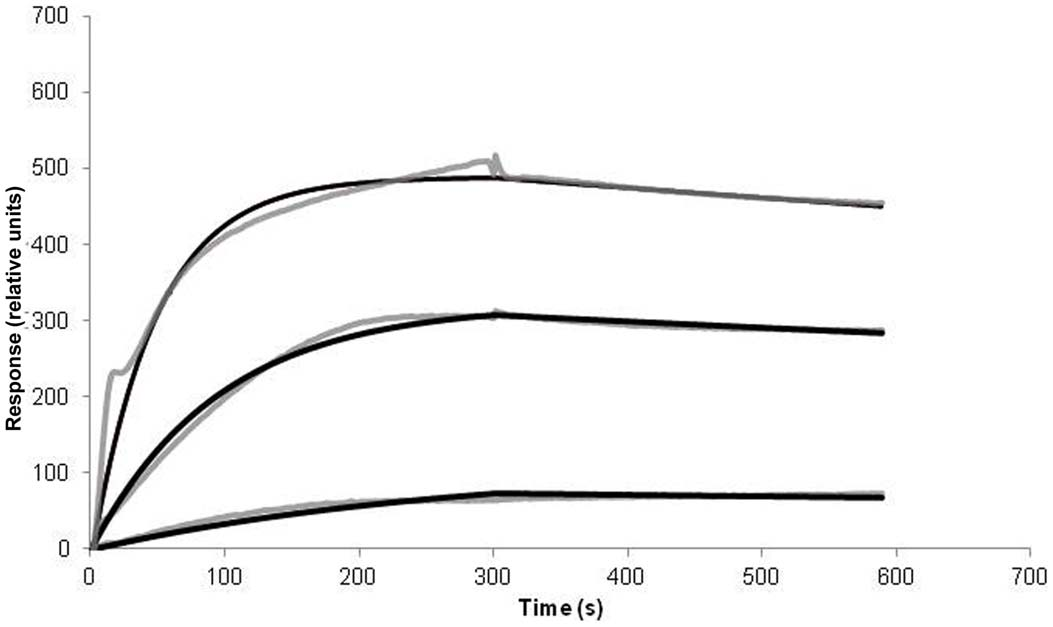
Binding of RI-OR2-TAT to immobilized Aβ42 fibrils, as shown by surface plasmon resonance (SPR) spectroscopy. The peptide was injected in solution at three different concentrations, for 5 mins at a flow rate of 30 µl/min. The upper line shows data for 6 µM RI-OR2-TAT, the middle line for 3 µM RI-OR2-TAT and the lower line for 1 µM RI-OR2-TAT. The non-specific binding obtained from the reference surface has been automatically subtracted from all data. Fitted curves are shown in black.

RI-OR2-TAT was shown to bind to immobilized Aβ in a concentration-dependent manner, when surface plasmon resonance (SPR) spectroscopy was used to estimate the binding constant between this inhibitor and fibrils derived from Aβ42 (**Fig. 2**). The binding was characterized by a fast association rate and a slow dissociation rate, with k_on_ values of 3680±615 M^−1^ s^−1^ and k_off_ values of 3.7±1.5×10^−4^ s^−1^, respectively. The curves were fitted separately using the simplest Langmuir 1∶1 interaction model, and the calculated apparent affinity value (k_d_) was 58–125 nM. This range of values represents a k_d_ of RI-OR2-TAT for Aβ42 fibrils that is ∼100-fold lower than that previously reported for RI-OR2 [16], indicating a correspondent increase in affinity. The fit using the 1∶1 Langmuir interaction model was considered satisfactory since the residuals automatically calculated from Qdat were maintained at <5% of the maximum experimental response [Qdat Tutorial, ICx Nomadics]. Using a “two-compartment” model, taking into account a possible mass transport limitation as a factor that influences the binding, we did not obtain better results.

### RI-OR2-TAT Inhibits the Toxic Effects of Aβ42 on Cultured Cells

**Figure 3:**
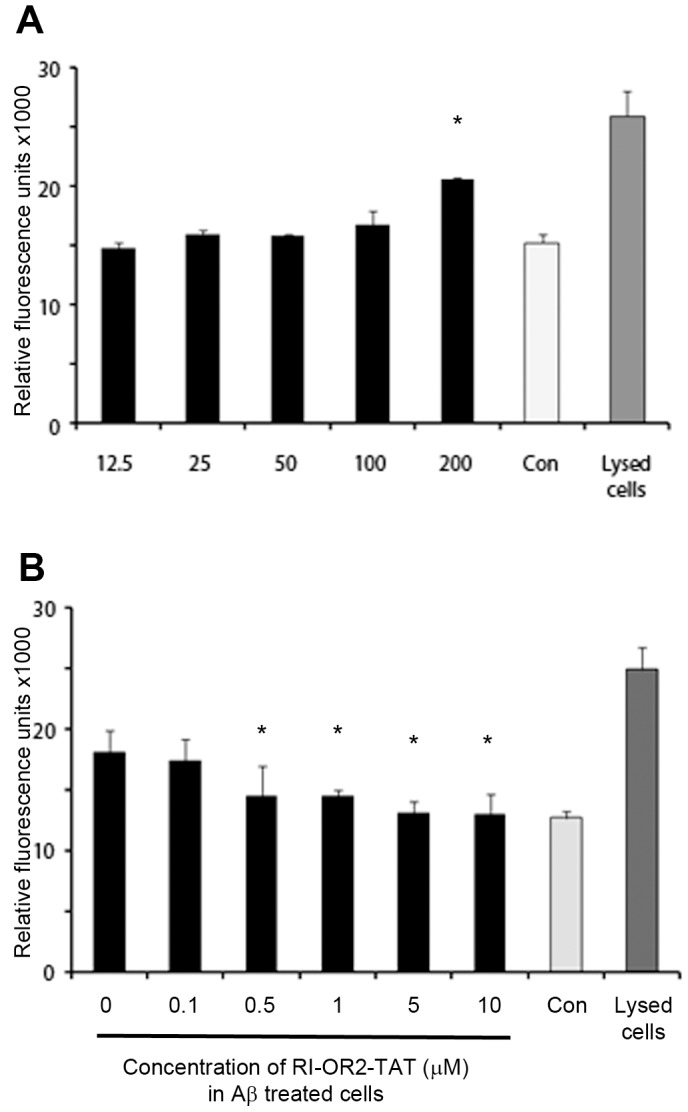
RI-OR2-TAT inhibits the toxic effects of Aβ42 on cells. (**A**) The black bars show data for the viability of SHSY-5Y neuroblastoma cells, as measured by LDH assay, following exposure to 12.5, 25, 50, 100 and 200 µM RI-OR2-TAT alone for 24 h. The light grey ‘Con’ bar shows data for cells maintained under the same conditions, but without RI-OR2-TAT, and the dark grey bar shows data for lysed cells. (**B**) Shows the ability of RI-OR2-TAT to protect against Aβ42-mediated toxicity. The black bars are LDH assay data for cells grown in the presence of 5 µM Aβ42 plus 0.1, 0.5, 1, 5 or 10 µM RI-OR2-TAT for 24 h. The ‘0’ bar shows data for cells grown in the presence of Aβ42 alone and the bars labelled ‘Con’ and ‘Lysed cells’ are the same as for (**A**). For both (**A**) and (**B**) *indicates p<0.05.

Exposure to RI-OR2-TAT for 24 h had no effect on the viability of SHSY-5Y cells, as measured by lactate dehydrogenase (LDH) assay, at concentrations of up to 100 µM, but the inhibitor was toxic at 200 µM (**Fig. 3A**). RI-OR2-TAT (at 0.5, 1, 5 or 10 µM) protected against the toxic effects of pre-aggregated Aβ42 (5 µM) on these cells (**Fig. 3B**).

### RI-OR2-TAT Enters Cultured Cells and Crosses the Blood Brain Barrier of APP/PS1 Transgenic Mice

**Figure 4:**
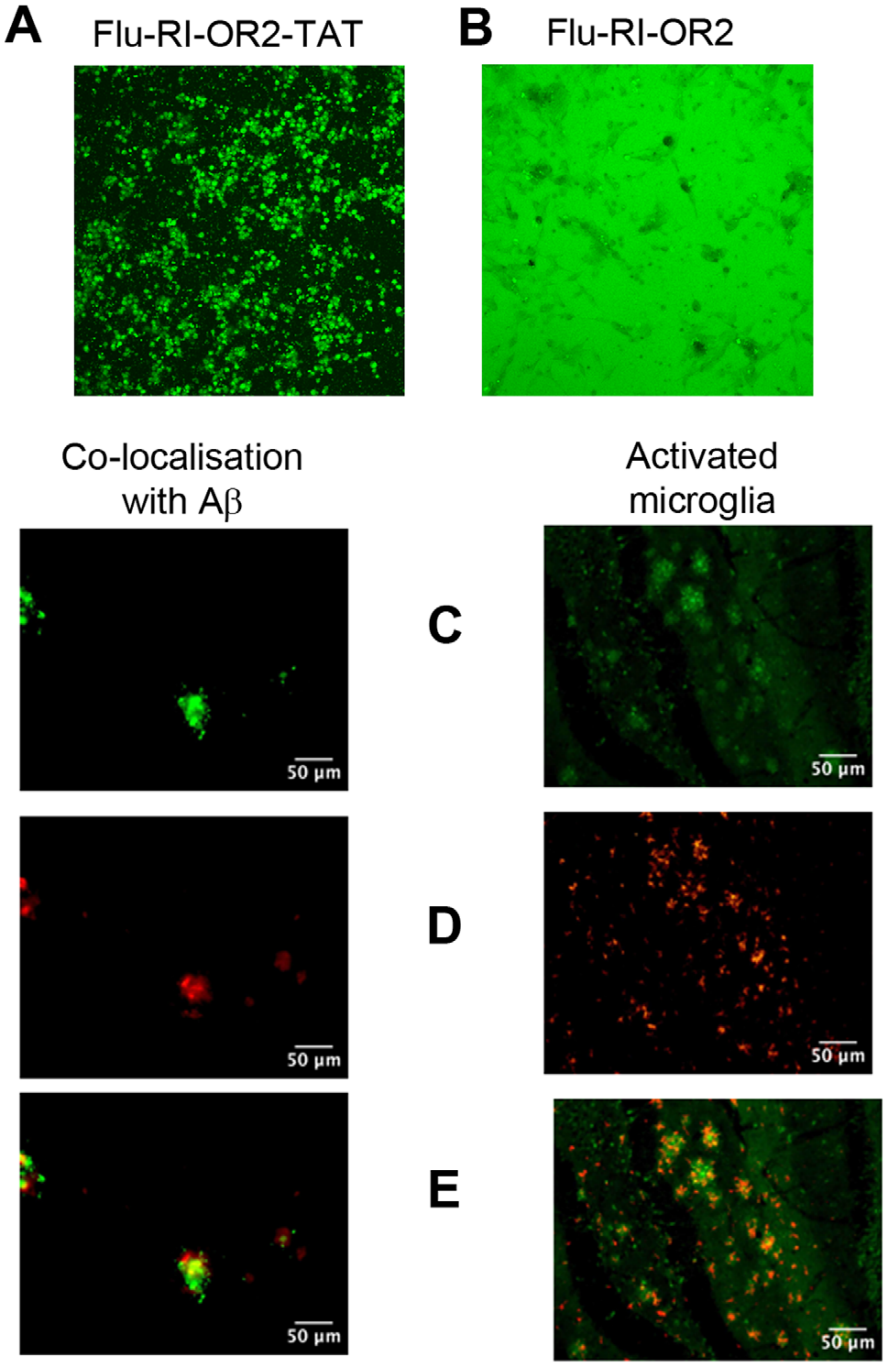
Flu-RI-OR2-TAT enters cultured SHSY-5Y cells and crosses the blood brain barrier of APP/PS1 transgenic mice. (**A**) Fluorescent microscope image of SHSY-5Y cells exposed to 1 µM RI-OR2-TAT for 10 mins. (**B**) Corresponding image for cells exposed to Flu-RI-OR2, also at 1 µM, but for 1 h. (**C**) Fluoresence images showing the detection of Flu-RI-OR2-TAT in sections of brain tissue following i.p. injection in 17-month old APP/PS1 transgenic mice. (**D**) Shows the same sections as in (**C**) but with detection using AlexaFluor 555 labelled antibody against either (on the left) Aβ or (on the right) activated microglia/Iba 1. (**E**) Shows merged images of (**C**) and (**D**) above. Flu-RI-OR2-TAT is seen to be co-localised with Aβ and with activated microglial cells.

Fluorescein-labelled versions of RI-OR2 (Flu-RI-OR2, fluorescein-rGffvlkGr-NH_2_) and RI-OR2-TAT (Flu-RI-OR2-TAT, fluorescein-rGffvlkGrrrrqrrkkrGy-NH_2_) were used for these experiments. Fluorescent and light microscope images of SHSY-5Y cells exposed to 1 µM Flu-RI-OR2-TAT for 10 mins showed the build up of fluorescence inside the cells (**Fig. 4A**), whereas the fluorescence associated with Flu-RI-OR2 remained in the culture medium (**Fig. 4B**). Flu-RI-OR2-TAT was detected in sections of brain tissue following intraperitoneal (i.p.) injection in 17-month old APP/PS1 transgenic mice, demonstrating that it can cross the BBB. In a double labelling study, using AlexaFluor 555 labelled antibody against Aβ, Flu-RI-OR2-TAT was found to be co-localised with amyloid plaques present in the cerebral cortex. In a parallel study AlexaFluor 555 labelled antibody targeting activated microglia/Iba 1 and Flu-RI-OR2-TAT were also found inside activated microglial cells (**Fig. 4C, D, E**).

### RI-OR2-TAT Decreases Brain Aβ Oligomer Levels and Amyloid Plaque Load in APP/PS1 Transgenic Mice

**Figure 5:**
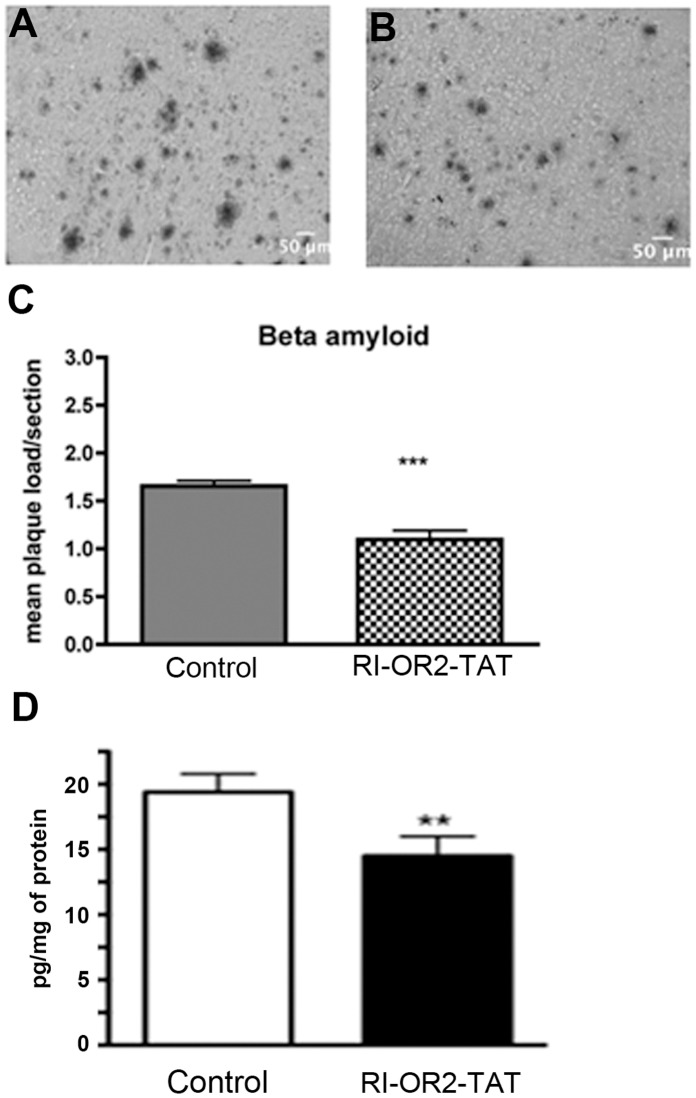
RI-OR2-TAT reduces the β-amyloid plaque load and levels of Aβ soluble oligomers in the brains of APP/PS1 transgenic mice. Representative images show amyloid deposits in the cortex region of 10 months old APP/PS1 mouse brains as shown by β-amyloid immunostaining in animals treated with (**A**) 0.9% saline or (**B**) 100 nmol/kg RI-OR2-TAT in 0.9% saline. (**C**) Quantitative analysis shows a decrease in mean plaque load in the cortex of APP/PS1 mice treated with RI-OR2-TAT compared to animals treated with saline. (**D**) Levels of soluble Aß oligomers in these brains. Values represent mean ± SEM of 4 animals per group, where *** p<0.0001; ** p<0.01, unpaired Student t-test.

As shown in **Fig. 5** **(A, B, C)**, treatment with 100 nmol/kg RI-OR2-TAT over a period of 21 days reduced amyloid plaque load by 32% in the cortex region of 10 months old APP/PS1 mouse brains, compared to animals treated with saline, as detected by β-amyloid immunostaining (p<0.0001, unpaired Student t-test, N = 4 animals per group). Soluble Aß oligomer levels in the brain, as detected by sandwich ELISA, were also reduced, by 25% (p<0.01, unpaired Student t-test) (see **Fig. 5D**).

### RI-OR2-TAT Decreases Microglial Activation and Oxidative Stress, and Stimulates Neurogenesis in the Brains of APP/PS1 Transgenic Mice

We assayed three markers to show the effect of RI-OR2-TAT on mouse brain: Iba1 is specifically expressed in microglia and is upregulated when the cells are activated [31]; 8-oxo-guanine is one of the most common DNA damage products seen in the presence of ROS; Doublecourtin is a microtubule-associated protein, expressed in immature neurons, that is required for migration into the cerebral cortex [32].

**Figure 6:**
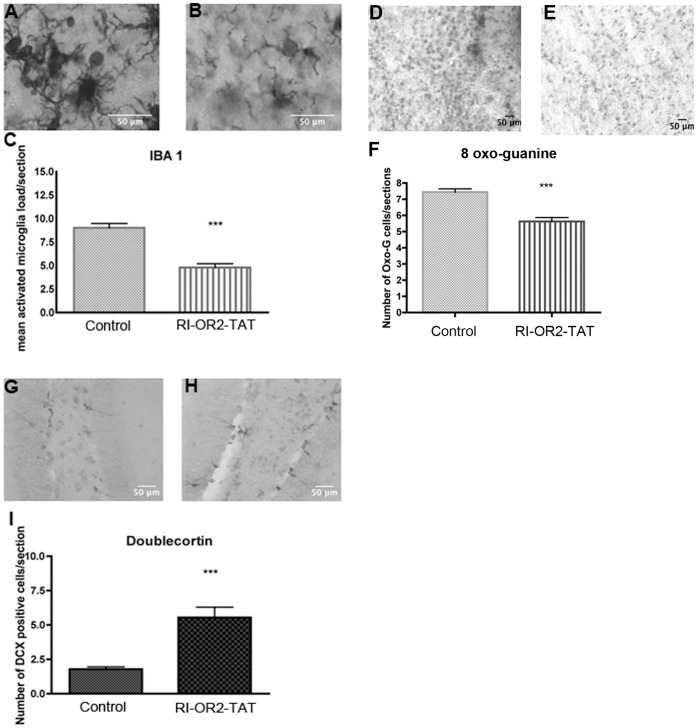
RI-OR2-TAT reduces the brain load of microglia and oxidative damage and stimulates neurogenesis in APP/PS1 transgenic mice. Representative images of activated microglia in the cortical region of APP/PS1 mouse brains as shown by Iba1 immunostaining in animals treated with (**A**) 0.9% saline or (**B**) 100 nmol/kg RI-OR2-TAT in 0.9% saline; (**C**) quantitative analysis shows a decrease in mean microglial load in APP/PS1 mice treated with RI-OR2-TAT; detection of 8 oxo-guanine in the brains of animals treated with (**D**) 0.9% saline or (**E**) 100 nmol/kg RI-OR2-TAT; (**F**) quantitative analysis shows a decrease in mean 8 oxo-guanine staining in APP/PS1 mice treated with RI-OR2-TAT; detection of immature neurons as stained for doublecortin in the dentate gyrus of APP/PS1 mice treated with (**G**) 0.9% saline or (**H**) 100 nmol/kg RI-OR2-TAT; (**I**) quantitative analysis shows an increase in mean doublecortin staining in APP/PS1 mice treated with RI-OR2-TAT. Values represent mean ± SEM of 4 animals per group, where *** p<0.0001, unpaired Student t-test.

Treatment with RI-OR2-TAT reduced the number of activated microglia observed in random sections of the cortex of APP/PS1 mouse brains of 10 months of age by 44% as shown by Iba1 immunostaining (p<0.0001, unpaired Student t-test) (**Fig. 6A, B, C**). This treatment also reduced the cortical level of 8-oxo-guanine immunostaining by 25% (p<0.0001, unpaired Student t-test) (see **Fig. 6D, E, F**), and there was an increase in the mean number of young doublecortin-expressing neurons in the dentate region by 210% (p<0.0001, unpaired Student t-test), all in comparison to control/saline treated animals (see **Fig. 6G, H, I**).

## Discussion

RI-OR2, without the TAT amino acid sequence, has been shown previously to inhibit Aβ oligomer and fibril formation *in vitro*, as demonstrated by the use of several different experimental techniques, and to block the toxic effects of Aβ on cultured cells [16]. Also, being a retro-inverso peptide, RI-OR2 is highly resistant to proteolysis and so should be stable *in vivo* [16]. Here, we have attached a retro-inverted version of the HIV-1 ‘TAT’ sequence to RI-OR2, in an attempt to target this inhibitor into the brain. The TAT sequence was incorporated in this way so that the protease resistance of the whole molecule would be maintained, which should result in good *in vivo* bioavailability. The data presented in **Fig. 1** and **Fig. 3** are consistent with our previous publication [16] and show that RI-OR2-TAT has similar properties to RI-OR2 as an Aβ aggregation inhibitor *in vitro*.

The retro-inverted TAT sequence was effective as a transit peptide, as demonstrated by the rapid entry of Flu-RI-OR2-TAT into cultured cells (this happened within minutes under our experimental conditions), whereas Flu-RI-OR2 stayed mainly in the culture medium (**Fig 4** **A, B**). Flu-RI-OR2-TAT was also able to penetrate across the BBB following its peripheral (i.p.) administration into APP/PS1 transgenic mice. This is not surprising because the TAT sequence has been employed previously to enable a variety of molecules to cross the BBB [33], [34] and this activity is known to be retained in retro-inverso TAT [35], [36]. Following penetration into the brain, Flu-RI-OR2–TAT was found to be attached to amyloid plaques and to activated microglial cells (**Fig. 4C, D, E**). This interaction with amyloid plaques would be anticipated given the relatively high binding affinity (k_d_ = 58–125 nM) between RI-OR2–TAT and Aβ42 amyloid fibrils, as calculated from our SPR data (**Fig. 2**). The marked increase in the binding affinity of RI-OR2–TAT compared to that previously reported for RI-OR2 [16] could be due to the presence of several positively charged amino-acid residues on the TAT portion of the retro-inverso peptide. In fact, Zhang *et al* [**37**], when studying the interaction of a series of synthetic peptides with Aβ, suggested that an increase in positive charge might increase their avidity for binding to amyloid fibrils. The finding that Flu-RI-OR2-TAT accumulates inside activated microglial cells is also expected, because they actively take up amyloid by phagocytosis and are involved in the clearance of Aβ [38]. Thus, Flu-RI-OR2-TAT within microglia is presumably attached to Aβ fibrils, although ultrastructural studies (e.g. immunogold labelling) would be required to confirm this.

In order to determine effects on brain pathology, RI-OR2-TAT was injected peripherally (i.p.) into APP/PS1 mice every day for 21 days, at 100 nmol/kg. This dose was chosen because it is similar to that used in a previous study with another peptide drug, which was also injected i.p. and reduced amyloid plaque count and inflammation in the brain [39]. When the brains were removed and examined, we found a marked and highly significant reduction in: (i) amyloid plaque load; (ii) the numbers of activated microglial cells; and (iii) the amount of oxidative damage (i.e. 8-oxo-guaninine staining). RI-OR2–TAT also reduced the level of Aβ oligomers in the cerebral cortex of the APP/PS1 mice. Further studies would be required to determine if any particular type of oligomer (e.g. dimer, trimer or higher molecular weight oligomer) is differentially affected, but our *in vitro* studies suggest that the inhibitor intervenes at a very early point in the aggregation pathway. These effects of RI-OR2–TAT on the level of Aβ oligomers (and on the other measures of brain pathology) might be improved by the use of an alternative dosing regimen, and/or by optimisation of the peptide sequence. We are also pursuing an alternative approach to increase the potency of RI-OR2-TAT against Aβ aggregation by attaching it onto the surface of nanoparticles, to produce a multivalent inhibitor. Multivalency has already been shown to improve the activity of another peptide aggregation inhibitor [40], and we have recently employed a similar strategy for curcumin, an alternative inhibitor of Aβ oligomer formation [41].

The inhibition of amyloid plaque load seen in the APP/PS1 transgenic mice injected with RI-OR2-TAT would be expected to result in fewer activated microglial cells, because the latter are known to congregate around the amyloid cores of senile plaques. The reduction of oxidative stress in the transgenic mice treated with RI-OR2-TAT could also be explained by its ability to reduce microglial cell numbers, because these cells are responsible for the formation and release of free radicals and cytokines involved in chronic inflammation and oxidation reactions [42]. However, the inhibition of oxidative damage seen in the treated mice could also be linked directly with the effects of RI-OR2-TAT on Aβ aggregation. Reactive oxygen species (ROS) have been shown to be generated during the early stages of Aβ aggregation, *via* an interaction between Aβ and redox-active metal ions, and an early-stage aggregation inhibitor would be expected to block this source of ROS formation [43]. Aβ oligomers have also been reported to induce calcium ion influx into cells and, subsequently, oxidative damage, through their ability to form ion-permeable ‘pores’ in cell membranes [44]. The reduction of microglial cell load and 8-oxo-guanine levels seen following treatment with RI-OR2-TAT are, therefore, both likely to be a ‘downstream’ consequence of the ability of this inhibitor to reduce Aβ oligomer and/or amyloid fibril formation. Our data, therefore, support the notion that Aβ aggregation is an earlier event than oxidative stress in the pathogenesis of AD, rather than the other way around [45].

The dramatic effect of treatment with RI-OR2-TAT on the number of immature neurons in the dendate gyrus of the APP/PS1 transgenic mouse brains was less anticipated, and suggests that the drug promotes a recovery of neurogenesis, although this will have to be confirmed by probing the rate of nerve cell proliferation using markers such as BrdU or phosphorylated histone-3. Inhibition of nerve stem cell proliferation could be a downstream consequence of Aβ aggregation. It is well known that pro-inflammatory cytokines reduce stem cell proliferation, and that this mechanism has serious consequences for brain function [46], [47]. Thus, one possibility is that RI-OR2-TAT could rescue brain stem cells from the damaging pro-inflammatory effects of Aβ. Another possibility is that Aβ oligomers have a direct toxic effect on brain stem cells and that RI-OR2-TAT protects against this effect.

There have been some high profile failures of various drug candidates targeted at the formation or aggregation of Aβ in recent years [48]. Most notably, the development of inhibitors of β-secretase (β-amyloid cleaving enzyme-1 or BACE-1) has proved to be difficult because of inherent medicinal chemistry problems [49] and inhibitors of γ-secretase have resulted in undesirable side-effects, due to inhibition of Notch processing [50]. ‘Notch sparing’ γ-secretase inhibitors are in development, but could fail because of side effects due to the unavoidable accumulation of the toxic carboxyl-terminal fragment of APP (CTFβ) [50]. Direct immunisation with Aβ, or passive immunization with anti-Aβ antibodies, is one of the most advanced approaches, but the first clinical trial of immunisation with pre-aggregated Aβ42 (AN1792) was stopped because of adverse effects involving detrimental T-cell-mediated brain inflammation [51]. Follow-up strategies to this are underway, but none have succeeded so far in any late-stage clinical trial [51]. This includes Bapineuzumab (Johnson and Johnson), which showed no significant effect on AD patients (results late 2012), and Solanezumab (Eli Lilly), which did not achieve key Phase III trial goals of improved cognition and function in mild-to-moderate AD sufferers (presented at CTAD 2012). Another promising discovery has been the finding that curcumin, derived from the dietary spice turmeric, is an effective inhibitor of Aβ aggregation [5], [52]. However, curcumin itself exhibits poor bioavailability and so is unsuitable as a drug candidate. Various derivatives of curcumin, with improved bioavailability, are being developed, but have not yet reached clinical trials [41], [53]. Because of these various problems and set-backs, there is still an urgent need for the development of alternative drugs for testing against AD. Recently, oral administration of the anti-cancer drug bexarotene has been shown to enhance clearance of soluble Aβ, through apoE-mediated mechanisms, and to reduce β-amyloid plaque deposits in mice, but is yet to be tested against human AD [54]. Apart from curcumin [52], the only other inhibitors of Aβ oligomer formation reported to have been tested in animal models of AD are 1,4-naphthoquinon-2-yl-L-tryptophan (NQTrp), which reversed phenotypic changes in *Drosophila* [55], and an amyloid-β42 oligomer-specific monoclonal antibody (A8), which improved memory performance in SAMP8 mice [56]. The data presented here for RI-OR2-TAT are encouraging and identify oxidative damage, inflammation, and inhibition of neurogenesis, as downstream consequences of Aβ aggregation. We hope to develop this inhibitor to the stage where it can enter human clinical trials.
