# Correction: A Novel Retro-Inverso Peptide Inhibitor Reduces Amyloid Deposition, Oxidation and Inflammation and Stimulates Neurogenesis in the APPswe/PS1ΔE9 Mouse Model of Alzheimer’s Disease

**DOI:** 10.1371/annotation/57e0a947-8600-4658-b04c-cf7a45c8bd8d

**Published:** 2013-09-20

**Authors:** Vadivel Parthsarathy, Paula L. McClean, Christian Hölscher, Mark Taylor, Claire Tinker, Glynn Jones, Oleg Kolosov, Elisa Salvati, Maria Gregori, Massimo Masserini, David Allsop

The seventh sentence of the sixth paragraph in the Discussion section should have cited references 41 and 52 instead of 5 and 52.

The correct sentence should read: Another promising discovery has been the finding that curcumin, derived from the dietary spice turmeric, is an effective inhibitor of Aβ aggregation [41], [52]. 

